# Level of and factors associated with awareness of gestational diabetes mellitus among pregnant women attending antenatal care at Kawempe National Referral Hospital: a cross sectional study

**DOI:** 10.1186/s12884-021-03927-x

**Published:** 2021-06-30

**Authors:** Elizabeth Byakwaga, Musa Sekikubo, Annettee Nakimuli

**Affiliations:** Department of Obstetrics and Gynaecology, School of Medicine, Makerere University College of Health Sciences, Kampala, Uganda

**Keywords:** Gestational Diabetes Mellitus, Uganda, Africa, Awereness

## Abstract

**Background:**

The burden of Gestational diabetes mellitus (GDM) is significantly increasing worldwide and the disorder causes substantial short term and long-term adverse effects both to the mother and the unborn baby. Public health measures to increase awareness of GDM among pregnant women may aid in prevention of the disease through life style modification, screening, early diagnosis and management but very few studies have assessed awareness of GDM among pregnant women in sub Saharan Africa and none of these are from Uganda. This study therefore sought to evaluate the level of and factors associated with awareness of GDM among pregnant women attending antenatal care at Kawempe National Referral Hospital (KNRH), the busiest obstetric unit in Uganda, so as to assess their health sensitization needs.

**Methods:**

This was a cross-sectional study. We recruited 403 participants at 30 weeks of gestation and above after giving written informed consent. Systematic sampling was used to select participants and data was collected using pretested interviewer-administered questionnaires. The collected data was entered in Epidata version 4.2 and exported to Stata for analysis. Continuous variables were summarized using mean and standard deviation. Categorical variables were summarized using frequencies and proportions. Factors associated with awareness were assessed at both bivariate and multivariate levels.

**Results:**

Four hundred three pregnant women were recruited, majority (35.5 %) were between 20 and 24 years and their mean age was 26.6 years. Only 125 (31 %) participants were aware of GDM. Age and educational level were significantly associated with awareness of GDM. Women aged 35 years and above were more likely to be aware of GDM (OR = 2.34 (95 % CI = 1.14–4.81) *p* = 0.021. Women with primary education or no education were less likely to be aware (OR = 0.48 (CI 0.24–0.96) *p* = 0.038.

**Conclusions:**

Awareness of GDM was poor among study participants. There is need to improve the health education programs in order to increase awareness of GDM among women attending ANC at KNRH. Women below 35 years of age and those with primary education or less should be specifically targeted when giving health education sessions so as to increase their awareness of GDM.

**Supplementary Information:**

The online version contains supplementary material available at 10.1186/s12884-021-03927-x.

## Background

The occurrence of diabetes mellitus for the first-time during pregnancy, also known as gestational diabetes mellitus (GDM), has been described by the International Diabetes Federation as a severe and neglected threat to maternal and child health [1]. In 2017, globally, 21.3 million live births had some form of hyperglycemia in pregnancy and of these, an estimated 84 % were due to GDM. Most of these cases of hyperglycemia in pregnancy reported were mainly in low- and middle-income countries (LMICs), where the human resource for health is not well equipped to handle such conditions and where access and utilization of maternal health services is still quite low [1]. In Africa, the prevalence of GDM has been estimated to range between 3.2 and 14 % depending on the diagnostic criteria used [2]. In Uganda, GDM is screened using an oral glucose tolerance test (OGTT) where GDM is diagnosed if one or more of the following criteria were met following a 75 g oral glucose ingestion; fasting plasma glucose of 5.1–6.9 mmol/L or 1 h plasma glucose ≥ 10.0 mmol/L or 2-hour plasma glucose 8.5–11.0 mmol/L. A study done in Uganda at St. Francis Nsambya Hospital revealed an alarmingly high prevalence of hyperglycemia first detected in pregnancy of 31.9 and 23.8 % of these had no risk factors [3].

Women with GDM are at greater risk for adverse pregnancy outcomes, notably, macrosomia of their newborns and pre-eclampsia [4, 5]. They are also over 20 times more likely to develop type 2 diabetes (T2DM), 2.8 times more likely to develop ischemic heart disease, and twice as likely to develop hypertension in their life time [6]. Furthermore, their infants are also at risk of becoming overweight or obese as young children and are more prone to developing T2DM [7, 8]. Despite the many complications associated with GDM, majority of women in LMICs are either not screened or improperly screened for GDM irrespective of the fact that these countries account for 80 % of the global diabetes mellitus burden as well as 90 % of all cases of maternal and perinatal deaths and poor pregnancy outcomes [9].

In Kawempe National Referral Hospital (KNRH), universal or even selective testing are not routinely done for pregnant women yet according to the International Federation of Gynecology and Obstetrics (FIGO) Initiative on gestational diabetes mellitus, GDM should be treated with a public health focus to raise awareness of the links between hyperglycemia and poor maternal and fetal outcomes as well as to the future health risks to mother and offspring and that all pregnant women should be tested for GDM [9]. The low screening for GDM may be majorly due to health systems policies that have not adopted the recommendation of universal screening but could also partly be due to lack of awareness about GDM and negative perceptions from mothers. Awareness of GDM among pregnant women could be one of the key strategies in primary prevention of the disease since it translates to adoption of healthy life styles, better health seeking behaviors including early screening, diagnosis and management of the disease. A study done in South Tamil Nadu, India, 88.7 and 51.2 % of women aware of GDM from urban and rural areas respectively believed that screening for GDM was essential [10]. Another study conducted in South Korea that aimed at measuring the effect of health promotion on diagnosis and management of diabetes mellitus showed that health promotion campaigns can enhance early diagnosis and management of diabetes mellitus [11]. These studies confirm the importance of awareness in disease prevention, early screening, diagnosis and management.

Very few studies have assessed awareness of GDM among pregnant women and none of these are from Uganda. This study therefore sought to evaluate the level of and factors associated with awareness of GDM among pregnant women attending antenatal care (ANC) at KNRH, which is the busiest obstetric unit in Uganda, so as to assess their health sensitization needs.

## Methods

This was a cross-sectional study conducted at Kawempe National Referral Hospital (KNRH). KNRH is located about 12kms away from the city centre of Kampala. The hospital is a women’s and children’s hospital and receives patients referred from other health facilities within and outside Kampala district, as well as walk-ins from the surrounding districts of Kampala and Wakiso. The catchment area of the hospital has a heterogenous population, consisting of the urban poor and those with average income. Pregnant mothers are expected to attend a minimum of eight ANC visits during the whole duration of pregnancy. The hospital runs an antenatal care (ANC) clinic 3 times a week with an estimated average daily attendance of 200–300. The study was conducted for 8 weeks during the months of January and February 2020.

This was an exploratory study that utilized a sample size of 403. Systematic sampling was used to select participants and every 15th eligible pregnant woman was selected. The first participant was chosen by considering the first pregnant woman that arrived at the ANC clinic and met the study criteria.

The study included pregnant women at 30 weeks of gestation and above calculated using dates of the last normal menstrual period (LNMP) or ultrasound scan where LNMP was unknown. All participants gave written informed consent to participate and pregnant women with preexisting history of type 1 or type 2 diabetes mellitus were excluded.

Data was collected using interviewer administered questionnaires adapted from previously published work [12]. The variables that were added in the questionnaire included history of antenatal screening for RBS, history of screening for OGTT, history of high blood pressure and HIV status of the participants. Questions assessing awareness of GDM were not changed. This questionnaire was piloted on women attending the antenatal clinics at Kawempe Hospital before use on study participants. The tool investigated the background information of the patient, obstetric history, medical history and 15 questions on awareness of gestational diabetes including its risk factors, diagnosis, treatment and complications. The questions on risk factors assessed the awareness on risk of GDM in patients with obesity prior to pregnancy, excessive weight gain during present pregnancy, history of diabetes mellitus during previous pregnancies, and family history of diabetes. The options provided were yes, no, and I don’t know. ‘Yes’ was considered as the right response. Awareness on the course of GDM and its consequences to the unborn baby and mother was assessed by questions on whether GDM usually disappeared after delivery, whether women with GDM and children born to these women were at an increased risk for future T2DM and obesity. ‘Yes’ was considered to be the correct response. To assess awareness on screening and diagnosis of GDM, questions on the test used and the timing of the test during pregnancy were asked. Options for the test used were urine test, blood test, blood test after a glucose load and I don’t know. Blood test and blood test after a glucose load were considered to be the correct answers. For the timing of the test the options given were, 12–16 weeks (3–4 months), 24–28 weeks (6-7months), during delivery and I don’t know. 24-28weeks (6–7 month) was considered as the correct answer. Their awareness on treatment of GDM was assessed using questions with options as diet and exercise, oral antidiabetic drugs, insulin injections and I don’t know. Diet and exercises, insulin injections, and oral antidiabetic drugs were considered as the correct response. Each correct response was given a score of 1 and each woman was scored out of a total of 15. A score of 0–5 was considered as poor knowledge, 6–9 as fair, and 10–15 as good knowledge of GDM. Patients found to have good and fair knowledge were considered to be aware of GDM and those with low knowledge were considered unaware.

Frequencies and percentages for the different categories of the variables were obtained through tabulating the respective variables. Cross tabulations of the outcome variable and each independent variable was done to obtain frequencies and percentages of the pregnant women who were aware and those who were not aware of GDM. Bivariable analysis was done using the simple logistic regression model to obtain crude odds ratios, 95 % confidence interval and the corresponding *p*-values. Multiple logistic regression using the Backward Elimination Model Building Technique was conducted to obtain adjusted odds ratios, 95 % confidence interval and the corresponding *p*-values. Variables with the least significance based on the *p*-values were removed one by one at the different steps of the model building. Checking for goodness of fit of the model was done based on the Akaike Information Criteria (AIC). Significance of the independent variables was set at *p* < 0.05.

## Results

A total of 403 pregnant women were interviewed. From Table 1 majority of participants (35.5 %) were aged 20–24. Only 30 (7.4 %) respondents were in the age category of 15–19. 386 (95.8 %) reported to be married whereas only 17 (4.2 %) were not married. Secondary education was the highest level attained by majority of the respondents 253 (62.8 %). Among them, 222 (55.1 %) were employed whereas 181 (44.9 %) were not employed.

**Table 1:** Baseline socio-demographic characteristics of study participants

Characteristic	Frequencies *n* = 403	Percentages (%)
**Age** Mean (SD) = 26.56 (5.52)		
15–19	30	7.4
20–24	143	35.5
25–29	114	28.3
30–34	72	17.9
35 and above	44	10.9
**Marital Status**		
Married/partnered	386	95.8
Not married/partnered	17	4.2
**Education**		
None/Primary	77	19.1
Secondary	253	62.8
Tertiary/University	73	18.1
**Employment Status**		
Not Employed	181	44.9
Employed	222	55.1

From Table 2, majority 218 (54.1 %) of the participants had history of carrying 1–3 viable pregnancies. Only 22 (5.5 %) of the study participants had high blood pressure and 23 (5.7 %) reported to have been living with HIV. Among them, 55 (13.6 %) and 5 (1.2 %) reported to have had random blood sugar (RBS) and oral glucose tolerance test (OGTT) respectively. Only 38 (9.4 %) reported having had a family member with diabetes mellitus.

**Table 2:** Obstetric and medical history of study participants

Characteristic	Frequencies	Percentages (%)
**Obstetric History**		
** Parity**		
Nulliparous	136	33.7
1–3	218	54.1
4 and above	49	12.2
** History of antenatal screening for RBS**		
Yes	55	13.6
No	348	86.4
** History of antenatal screening for OGTT**		
Yes	5	1.2
No	398	98.8
** History of GDM in previous pregnancies** (***n*** = 278)		
Yes	20	7.2
No	258	92.8
**Medical History**		
** High Blood Pressure**		
Yes	22	5.5
No	381	94.5
** HIV**		
Yes	23	5.7
No	380	94.3
** Family Member with Diabetes Mellitus**		
Yes	38	9.4
No	365	90.6

Only 125 (31.0 %) of the participants were aware of GDM (Table 3). Table 4, shows the bivariate analysis for factors associated with awareness of GDM among pregnant women attending antenatal care at KNRH. At multivariable analysis as shown in Table 5, age and education were significantly associated with being aware of Gestational Diabetes Mellitus among pregnant women attending antenatal care at Kawempe National Referral Hospital. The odds of being aware of Gestational Diabetes Mellitus among pregnant women aged 35 and above are 2.34 [95 % CI = 1.14–4.81] times that of women aged 20–24. The odds of being aware of Gestational Diabetes Mellitus among pregnant women who attained primary education was 0.48 [95 %CI = 0.24–0.96] times that of pregnant women who attained secondary education. Pregnant women who had attained primary education were 52 % less likely to be aware of Gestational Diabetes Mellitus compared to those who had attained secondary education.

**Table 3:** Level of awareness of gestational diabetes mellitus

Dependent variable	Frequencies	Percentages (%)
**Level of awareness of Gestational Diabetes Mellitus (GDM)**		
Not aware of GDM	278	69.0
Aware of GDM	125	31.0

**Table 4:** Bivariate analysis for factors associated with awareness of GDM among pregnant women attending ANC at KNRH

Variables	Level of awareness of GDM		Crude OR	[95 % CI]	*p*-value
	**Aware n (%)**	**Not aware n (%)**			
**Age**					
20–24	39 (27.27)	104 (72.73)	Ref.		
15–19	5 (16.67)	25 (83.33)	0.53	[0.19–1.49]	0.231
25–29	32 (28.07)	82 (71.93)	1.04	[0.60–1.80]	0.887
30–34	28 (38.89)	44 (61.11)	1.70	[0.93–3.09]	0.084
35 and above	21 (47.73)	23 (52.27)	2.43	[1.21–4.89]	0.012
**Marital Status**					
Married	119 (30.83)	267 (69.17)	Ref.		
Not Married	6 (35.29)	11 (64.71)	1.22	[0.44–3.39]	0.698
**Education**					
Secondary	79 (31.23)	174 (68.77)	Ref.		
None/Primary	15 (19.48)	62 (80.52)	0.53	[0.29–0.99]	0.048
Tertiary/University	31 (42.47)	42 (57.53)	1.63	[0.95–2.78]	0.075
**Employment Status**					
Employed	79 (35.59)	143 (64.41)	Ref.		
Not Employed	46 (25.41)	135 (74.59)	0.62	[0.40–0.95]	0.029
**Parity**					
Nulliparous	40 (29.41)	96 (70.59)	Ref.		
1–3	66 (30.28)	152 (69.72)	1.04	[0.65–1.67]	0.863
4 and above	19 (38.78)	30 (61.22)	1.52	[0.77–3.01]	0.230
**History of antenatal screening for RBS**					
No	105 (30.17)	243 (69.83)	Ref.		
Yes	20 (36.36)	35 (63.64)	1.32	[0.73–2.40]	0.358
**History of antenatal screening for OGTT**					
No	123 (30.90)	275 (69.10)	Ref.		
Yes	2 (40.00)	3 (60.00)	1.49	[0.25–9.05]	0.665
**History of High Blood Pressure**					
No	116(30.45)	265(69.55)	Ref.		
Yes	9 (40.91)	13 (59.09)	1.58	[0.66–3.81]	0.306
**HIV**					
No	117(30.79)	263(69.21)	Ref.		
Yes	8 (34.78)	15 (65.22)	1.20	[0.49–2.91]	0.688
**Family Member with Diabetes Mellitus**					
No	111(30.41)	254(69.59)	Ref.		
Yes	14 (36.84)	24 (63.16)	1.33	[0.67–2.68]	0.417

**Table 5:** Multivariate analysis for factors associated with awareness of GDM among pregnant women attending ANC at KNRH

Variables	Adjusted OR	(95 % CI)	*p*-value
Age			
20–24	Ref.		
15–19	0.62	(0.21–1.83)	0.385
25–29	0.95	(0.53–1.70)	0.856
30–34	1.69	(0.90–3.15)	0.102
35 and above	**2.34**	**(1.14–4.81)**	**0.021**
Marital Status			
Married	Ref.		
Not Married	1.82	(0.64–5.14)	0.261
Education			
Secondary	Ref.		
None/Primary	**0.48**	**(0.24–0.96)**	**0.038**
Tertiary/University	1.37	(0.79–2.39)	0.261
Employment Status			
Employed	Ref.		
Not Employed	0.73	(0.46–1.15)	0.175
Parity			
Nulliparous	Ref.		
1–3	0.75	(0.43–1.32)	0.319
4 and above	0.69	(0.27–1.77)	0.438
History of antenatal screening for RBS			
No	Ref.		
Yes	1.35	(0.70–2.60)	0.372
History of antenatal screening for OGTT			
No	Ref.		
Yes	1.53	(0.11–21.81)	0.753
History of High Blood Pressure			
No	Ref.		
Yes	1.21	(0.42–3.50)	0.726
HIV			
No	Ref.		
Yes	0.97	(0.35–2.70)	0.959
Family Member with Diabetes Mellitus			
No	Ref.		
Yes	1.07	(0.51–2.24)	0.857

## Discussion

In this study, about one third (31 %) of the pregnant women were aware of GDM, its risk factors, complications and how it is screened and treated. These findings were very much in agreement with studies by other researchers who found awareness of GDM to be low among pregnant women attending antenatal clinics. Shriraam et al., in South India [12] and a study done in Bangladesh [13] found that only 17.5 and 26.3 % respectively of pregnant women were aware of GDM which was slightly lower when compared to this study and this could be because these studies included women from rural settings where as our study was conducted in an urban setting but with a heterogenous population consisting of the urban poor and those with average income. Similarly, a study done in Southern Nigeria [14] showed an overall awareness score of 26.2 % though this study assessed awareness of GDM among women of reproductive age and not pregnant women as ours. On the contrary, a study done in Sharjah, a city in the United Arab Emirates (UAE) showed that a greater proportion of women 73.5 % were aware of the disease [15]. Adequate awareness of GDM translates to early screening/diagnosis and better adherence to management strategies hence improving pregnancy outcomes. In a systematic review, increased patient understanding of the necessity and procedure of screening resulted in a higher screening uptake thus enabling earlier diagnosis and management of diabetes [16]. Additionally, a study done in China showed that health education combined with routine nursing care in pregnant women diagnosed with GDM significantly resulted in better pregnancy out comes such as lower incidence of premature delivery, polyhydramnios, postpartum hemorrhage, fetal distress and urinary tract infections [17].

Inadequate health care provider knowledge on GDM, late ANC attendance as well as lack of standard guidelines, supplies and equipment for screening and management of GDM are also major barriers to early detection and management of GDM in LMICs [18].

In this study, age of the participants was independently associated with awareness of GDM, with the older women more likely to be aware than the younger ones. The odds of being aware of GDM among pregnant women aged 35 years and above were 2.34 times that of women aged 20–24 years. Studies by Dhyani et al., Gastrich at al, Choi at al and Sangeetha at al showed similar findings with older participants (> 30 years) more likely to be aware of GDM than the younger ones [19–22]. This could be explained by the fact that most older women have a higher parity than the younger ones hence are more likely to have had exposure to information on GDM from ANC clinics attended in the previous pregnancies, but parity was not associated with increased awareness of GDM in our study. On the contrary, a study by Price et al. done in Samoa found that younger participants were more likely to be aware of the disease compared to older participants; that is women aged 18–22 years had the greatest level of awareness of GDM (61 %; *n* = 86), while those aged 33–37 years had the lowest level of awareness (39 %; *n* = 55) [23].

Level of education of the participants was significantly associated with awareness of GDM, with women who had attained primary education or less 52 % less likely to be aware of GDM compared to those who had attained secondary education. Bhowmik et al., Balaji et al. and Lakshmi et al. reported similar findings. In these studies, participants with higher educational status were found to have a significantly higher mean knowledge score than their counterparts [10, 13, 24]. This could be because education has a strong impact on health literacy; that is, pregnant women with higher education status are able to read health posters, get information from health-related articles and literature and have better understanding of the health information given to them by the health personnel during antenatal care. It is also possible that women with higher education status have better exposure to mass media like television, radio and internet through which they can gain health information and knowledge. Studies on effects of public health education campaigns through mass media showed that these platforms can evoke positive behavioral change [25–27] hence these media platforms can effectively act as channels through which the public can obtain information on GDM.

### Limitations of the study

The study only included pregnant women at 30 weeks of gestation and above hence excluding pregnant women that were in the first and second trimester, this was done in order to investigate if history of screening for GDM in the index pregnancy was associated with awareness of GDM. Screening for GDM is routinely done between 24 and 28 weeks of gestation. The awareness of GDM in women in the early gestation period may be different from those 30 weeks and above.

## Conclusions

A significant proportion of pregnant women were not aware of GDM, it’s risk factors, complications, how it is prevented, screened and treated. Increasing age and higher education were associated with increased awareness of GDM. There is therefore need to improve the health education programs both at community level and in health facilities in order to increase awareness of GDM among pregnant women in Uganda. Women below 35 years of age and those who have attained primary education or less should be specifically targeted when giving health education sessions so as to increase their level of awareness of GDM.

## Supplementary Information

**Additional file 1.**

## Data Availability

All data generated or analysed during this study are included in this published article [and its supplementary information files].

## Abbreviations

ANC: Antenatal care clinic
DM: Diabetes mellitus
GDM: Gestational diabetes mellitus
HIV: Human immunodeficiency virus
KNRH: Kawempe National Referral Hospital
LMICs: Low- and Middle-income countries
LNMP: Last Normal Menstrual Period
OGTT: Oral glucose tolerance test
RBS: Random blood sugar
T2DM: Type 2 diabetes mellitus
WHO: World Health Organization
