# Supplementary material for: Level of and factors associated with awareness of gestational diabetes mellitus among pregnant women attending antenatal care at Kawempe National Referral Hospital: a cross sectional study
Source: BMC Pregnancy Childbirth. 2021 Jun 30;21:467. doi: 10.1186/s12884-021-03927-x (PMC8247146; doi:10.1186/s12884-021-03927-x)
Supplement: Supplementary file 1 — Additional file 1. [file 12884_2021_3927_MOESM1_ESM.docx]

## **AWARENESS OF GDM QUESTIONNAIRE**

**Study title: Level of and factors associated with awareness of gestational diabetes mellitus among pregnant women attending antenatal care at Kawempe National Referral Hospital.**

Interviewer’s name………………………………………………………………

Participant’s study number…………………………….

Date; dd……mm……yr……….

**A. Socio-demographic characteristics of respondent**

1. Participant’s date of birth; dd.…..mm……yr……….
2. Participant’s age in years……
3. What is your marital status?

□ Married □ Divorced □ Widowed □ Single

1. Education level

□Primary □ Secondary □ Tertiary/university

□ None

1. Income

Do you have an income generating source?

□ YES □ NO

If yes, specify what it is.

……………………………………………………

**B. Obstetric related variables**

1. Gravidity ………………...
2. Parity ……………………
3. LNMP dd……mm……yr………
4. Gestational age in weeks

By LNMP…………….

By 1^st^ scan………………

1. Have you attended the ANC clinic before?

□YES □ NO

If Yes answer Q 6 and 7, if No go to Q8

1. At what gestation age (in weeks) did you start your ANC visits?

……………………………………

1. How many times have you attended ANC clinics?

□ One □ Two

□ Three □ More than three

1. Do you have a record of antenatal screening for any of the following? (Interviewer must check the ANC card for the following investigations)
2. HIV YES NO
3. Hepatitis B virus YES NO
4. Urinalysis YES NO
5. HB level YES NO
6. RBS YES NO
7. OGTT YES NO
8. Do you already have children?
9. Yes
10. No
11. Do you have history of gestational diabetes mellitus in previous pregnancies?
12. Yes
13. No
14. Don’t know
15. If yes to Q10 above, were there any birth complications? Tick all that apply.
16. None
17. Small size baby
18. Large size baby (> 4kgs)
19. Pre-eclampsia
20. Premature birth
21. Still birth
22. Other (please explain; include congenital anomalies)

………………………………………………………………..

**C. Medical factors**

1. Do you have high blood pressure?
2. Yes
3. No
4. Do you have cardiac disease?
5. Yes
6. No
7. Do you have renal disease?
8. Yes
9. No
10. Do you have a family member with diabetes mellitus? (Include immediate blood relatives only, not cousins or spouse/partner)
11. Yes
12. No
13. What is your HIV status?
14. Positive
15. Negative
16. If HIV positive, is she on HAART
17. Yes
18. No
19. If on HAART, what is the duration and regimen?

……………………………………………..

**D. Patient knowledge on gestational diabetes mellitus**

1. Can diabetes occur for the first time in pregnancy?
2. Yes
3. No
4. Don’t know

If yes answer Q 2 to 9 and part E

1. Do you think the following things increase the chances of a person to develop gestational diabetes?
2. Being overweight before getting pregnant
3. Yes
4. No
5. I don’t know
6. Gaining lots of weight during pregnancy
7. Yes
8. No
9. I don’t know
10. Past history of gestational diabetes
11. Yes
12. No
13. I don’t know
14. Family history of diabetes
15. Yes
16. No
17. I don’t know
18. Is Gestational Diabetes Mellitus harmful to the un-baby if not treated?
19. Yes
20. No
21. I don’t know
22. Does Gestational Diabetes Mellitus usually disappear after pregnancy?
    1. Yes
    2. No
    3. I don't know
23. Are women with Gestational Diabetes Mellitus at an increased risk of developing Type 2 Diabetes Mellitus in future?
    1. Yes
    2. No
    3. I don't know
24. What are the long-term health consequences for children born to GDM mothers?
    1. Weight gain
    2. Type 2 Diabetes Mellitus
    3. I don't know
25. How is Gestational Diabetes Mellitus screened?
26. Using a urine test
27. Using a blood test
28. Blood test after a glucose load
29. I don’t know
30. When should GDM be screened for?
31. 12-16 weeks (3-4 months)
32. 24-28 weeks (6-7months)
33. During delivery
34. I don’t know
35. How is Gestational Diabetes Mellitus treated? More than one box can be answered
36. Through diet and exercise
37. Insulin injections
38. Oral anti-diabetic drugs
39. I don’t know

**E. Patient’s source of information.**

- - - 1. What source(s) did you learn your answers to questions 1 to 9 from? Please tick all that apply.
  1. Friends/ neighbors
  2. Family
  3. TV/ radio
  4. Hospital charts/ posters
  5. Health care worker
  6. Doctor
  7. Newspapers/ magazines
  8. Other (please state) ……………………………………………

**F. Thank you very much for taking the time to complete this questionnaire.**
